# Shiga Toxin Receptor Gb3Cer/CD77: Tumor-Association and Promising Therapeutic Target in Pancreas and Colon Cancer

**DOI:** 10.1371/journal.pone.0006813

**Published:** 2009-08-28

**Authors:** Ute Distler, Jamal Souady, Marcel Hülsewig, Irena Drmić-Hofman, Jörg Haier, Alexander W. Friedrich, Helge Karch, Norbert Senninger, Klaus Dreisewerd, Stefan Berkenkamp, M. Alexander Schmidt, Jasna Peter-Katalinić, Johannes Müthing

**Affiliations:** 1 Institute of Hygiene, University of Münster, Münster, Germany; 2 Institute of Medical Physics and Biophysics, University of Münster, Münster, Germany; 3 Department of Pathology, Laboratory for Clinical and Forensic Genetics, University Hospital and Medical School Split, Split, Croatia; 4 Department of General Surgery, University Hospital Münster, Münster, Germany; 5 Sequenom GmbH, Hamburg, Germany; 6 Institute of Infectiology, University of Münster, Münster, Germany; Technical University Munich, Germany

## Abstract

**Background:**

Despite progress in adjuvant chemotherapy in the recent decades, pancreatic and colon cancers remain common causes of death worldwide. Bacterial toxins, which specifically bind to cell surface-exposed glycosphingolipids, are a potential novel therapy. We determined the expression of globotriaosylceramide (Gb3Cer/CD77), the Shiga toxin receptor, in human pancreatic and colon adenocarcinomas.

**Methodology/Principal Findings:**

Tissue lipid extracts of matched pairs of cancerous and adjacent normal tissue from 21 pancreatic and 16 colon cancer patients were investigated with thin-layer chromatography overlay assay combined with a novel mass spectrometry approach. Gb3Cer/CD77 was localized by immunofluorescence microscopy of cryosections from malignant and corresponding healthy tissue samples. 62% of pancreatic and 81% of colon adenocarcinomas showed increased Gb3Cer/CD77 expression, whereas 38% and 19% of malignant pancreas and colon tissue, respectively, did not, indicating an association of this marker with neoplastic transformation. Also, Gb3Cer/CD77 was associated with poor differentiation (G>2) in pancreatic cancer (P = 0.039). Mass spectrometric analysis evidenced enhanced expression of Gb3Cer/CD77 with long (C24) and short chain fatty acids (C16) in malignant tissues and pointed to the presence of hydroxylated fatty acid lipoforms, which are proposed to be important for receptor targeting. They could be detected in 86% of pancreatic and about 19% of colon adenocarcinomas. Immunohistology of tissue cryosections indicated tumor-association of these receptors.

**Conclusions/Significance:**

Enhanced expression of Gb3Cer/CD77 in most pancreatic and colon adenocarcinomas prompts consideration of Shiga toxin, its B-subunit or B-subunit-derivatives as novel therapeutic strategies for the treatment of these challenging malignancies.

## Introduction

Pancreatic and colon cancers are the fourth and second most frequent causes of cancer mortality in the Western world, respectively, accounting for estimated 84,250 deaths in 2008 in the U. S. alone [1]. With a median survival period of about 6 months and 5-year survival rates<5% pancreatic cancer ranks among the most lethal of the common tumors. The prognosis for patients suffering from colon carcinomas with distant metastasis at the time of diagnosis is almost as poor as for pancreatic cancer [2]. Only about 10–15% of the pancreatic cancer patients are candidates for potentially curative surgery [3], [4], underlining the urgent need to develop novel strategies to treat patients with these unresectable tumors. Targeted therapies, for example, based on bacterial and plant toxins or monoclonal antibodies, which recognize cell surface glycosphingolipids (GSLs), that are overexpressed in pancreas and/or colon cancer, might prove to be promising approaches for adjunct therapy after surgery [5]–[9].

GSLs, consisting of a hydrophilic oligosaccharide chain and a hydrophobic ceramide membrane anchor [10], are expressed as integral constituents of lipid rafts in the outer leaflet of the plasma membrane [11]. Besides their involvement in cell growth regulation and cell adhesion [12], cell surface-exposed oligosaccharide chains of GSLs “serve” as attachment sites for bacteria [13] and are exploited by different toxins, including Shiga toxins (Stxs), for surface binding, intracellular trafficking, and signalling events [14]. Stxs (also termed verotoxins), which are produced from pathogenic *Escherichia coli* strains [15]–[19], belong to the AB_5_ family of bacterial toxins. They consist of an enzymatically active A-subunit that inhibits protein biosynthesis by modifying host rRNA and a nontoxic homopentameric B-subunit [20], [21]. The B-pentamer binds to its preferential GSL receptor globotriaosylceramide (Gb3Cer/CD77) and triggers internalization of the AB_5_-Gb3Cer complex by receptor mediated endocytosis *via* clathrin-coated vesicles [22] or by endocytic routes that do not involve clathrin-coated pits [23]. The toxin-receptor complex undergoes retrograde transport through the Golgi network to the endoplasmic reticulum. After retro-translocation into the cytosol [24], one molecule of the proteolytically processed A_1_-subunit can inhibit protein synthesis and kill a cell.

Aberrant expression of GSLs occurs in almost all human and animal cancers [12], [25] and many tumor-associated antigens are now known to be GSLs. Increased expression of the Stx-receptor Gb3Cer/CD77 has been reported on various solid tumors such as ovarian [26] and breast cancer [27] or malignant meningioma [28]. Recently, enhanced expression of Gb3Cer/CD77 has been described as correlating with the development of metastasis in colon cancer [9], [29]. Gb3Cer/CD77 also enables tumor-specific plasma membrane localization of proteins (e. g. Hsp70) [30]. As cell surface molecules, tumor-associated GSLs are accessible to antibodies or GSL-binding toxins (i.e., Stx), making them candidate targets for oncological applications [31]. Consequently, the Stx-ligand Gb3Cer/CD77 is currently under investigation as a potential target candidate for toxin-based therapeutics [28], [29], [32], [33].

In this study we analyzed pancreatic and colon cancer tissue for the expression of Gb3Cer/CD77, using thin-layer chromatography (TLC) of GSL extracts from tissues and immunohistological staining of tissue cryosections. Enhanced expression of Stx-receptors was detectable in most pancreatic and colon cancer tissues, respectively, indicating an association with neoplastic transformation. Furthermore, structural modifications of the lipid anchors of tumor-associated Gb3Cer/CD77 species were detected in cancerous tissues of both tumor kinds employing a novel mass spectrometry approach combined with TLC immunodetection [8], [34]. Thus, enhanced expression and lipid alterations of Gb3Cer/CD77 in carcinomas render this target a promising candidate for Stx and/or Stx-constructs in oncological applications of both pancreas and colon malignancies.

## Materials and Methods

### Ethics Statement

The Local Ethical Committee of the Medical Council of Westfalen-Lippe and the Medical Faculty of the University Hospital Münster (Münster, Germany) approved the current study (reference number 1IXHai). All patients were informed and consented in writing.

### Surgical Specimens

The study was performed using samples of pancreatic (n = 21) and colon (n = 16) adenocarcinomas from patients who had undergone surgery for their primary tumors [5]. Tumor histology was determined following the criteria of the UICC [35]. The clinicopathologic characteristics of the cohort of patients with the carcinomas are described in Supplementary Tables S1 and S2. Tumor specimens were snap frozen in liquid nitrogen immediately after surgical intervention and stored at −80°C until analyzed. Corresponding control specimens were obtained from the same patient at the same organ site without macroscopic tumor involvement that was confirmed by histological examination.

### Preparation of Lipid Extracts from Surgical Specimens

Tissues were homogenized and extracted twice with 2 mL of chloroform/methanol (1/2, v/v), 2 mL of chloroform/methanol (1/1, v/v), and 2 mL of chloroform/methanol (2/1, v/v). The combined supernatants of each tissue extract (12 mL) were dried by rotary evaporation and phospholipids were saponified by incubation in 4 mL of aqueous 1 N NaOH for 1 h at 37°C. After neutralization with 400 µL of 10 N HCl, the samples were dialyzed against deionized water and dried by rotary evaporation. The extracts were adjusted to defined volumes of chloroform/methanol (2/1, v/v) corresponding to 0.1 mg wet weight per µL.

### Purification of Stx1

Stx1 was purified from *E. coli* C600(H19J) carrying the *stx_1_* gene from *E. coli* O26:H11 strain H19 [36]. Briefly, bacteria were disintegrated by sonication and extracted with polymyxin B (3000 U/ml) (Sigma-Aldrich Chemie GmbH, Taufkirchen, Germany). After centrifugation, the supernatant containing crude Stx1 was concentrated by ultrafiltration and subjected to chromatography on hydroxylapatite column followed by chromatofocusing [37]. The column fractions containing Stx1 were pooled, dialyzed and aliquots were stored at −70°C until use. Purity of the Stx1 preparation was monitored by SDS-PAGE, and the structural integrity of the toxin was checked by peptide mapping employing mass spectrometry [21]. Biological activity was determined by Vero cell cytotoxicity [38].

### Anti-Gb3Cer/CD77 and Anti-Stx1 Antibodies

The preparation and specificity of polyclonal chicken anti-Gb3Cer/CD77 antibody recognizing the Galα1-4Galβ1-4Glc-residue of Gb3Cer/CD77 species has been described [39], [40]. Stx1 was detected with the mouse IgG1 monoclonal anti-Stx1 antibody 109/4-E9b (Sifin, Berlin, Germany).

### High-performance Thin-layer Chromatography (TLC) and Reference GSLs

A mixture of neutral GSLs from human erythrocytes, consisting of monohexosylceramide, lactosylceramide, Gb3Cer/CD77, and globotetraosylceramide (Gb4Cer), was used as reference [40] for TLC antibody and Stx1 overlay assays. The nomenclature of GSLs follows the IUPAC-IUBM recommendations 1997 [41]. Reference GSLs and tissue lipid extracts were applied to glass-backed silica gel 60 precoated high-performance thin-layer chromatography (TLC) plates (no. 1.05633.0001; Merck, Darmstadt, Germany) with an automatic applicator (Linomat IV, CAMAG, Muttenz, Switzerland) and separated in chloroform/methanol/water (120/70/17, each by vol., with 2 mM CaCl_2_). Reference GSLs were stained with orcinol.

### TLC Overlay Assay

Gb3Cer/CD77 was detected using the overlaid polyclonal chicken antibody or Stx1 as described [40], [42]. Primary anti-Gb3Cer/CD77 and secondary alkaline phosphatase-labeled antibodies (Dianova, Hamburg, Germany) were used in 1∶2000 dilutions. Bound secondary antibodies were visualized by color development with 0.05% (w/v) 5-bromo-4-chloro-3-indolylphosphate p-toluidine salt (BCIP, no. 6368.3; Roth, Karlsruhe, Germany). The immunostained chromatograms were washed with glycine buffer and stored at −20°C. Blue immunopositive Gb3Cer/CD77-bands were quantified with a CD60 scanner (Desaga, Heidelberg, Germany, software ProQuant^R^, version 1.06.000). Gb3Cer/CD77 amounts were determined semiquantitatively as relative expression compared with reference GSLs. The secondary densitometric data (see “Statistical Analysis” below) were used to define 4 categories of tumor expression compared to the normal tissues by formal cut-point analysis: high overexpression (category I, 4000<x), moderate overexpression (category II, 700<x<4000), equal expression (category III, −700<x<700), and lowered expression (category IV, x<−700) for pancreas carcinoma and high overexpression (category I, 2000<x), moderate overexpression (category II, 200<x<2000), equal expression (category III, −200<x<200), and lowered expression (category IV, x<−200) for colon carcinoma, respectively.

### Immunohistochemistry

Immunocytochemical staining was conducted as described [8]. Briefly, cryosections (4 µm) were incubated with the anti-Gb3Cer/CD77 antibody (1∶15) and an anti-CD31 antibody (1∶200) (mouse IgG1, clone MEM-05, Immunotools, Friesoythe, Germany). Antibodies binding to Gb3Cer/CD77 and CD31 were detected with dichlorotriazinylamino fluorescein (DTAF)-labeled anti-chicken-IgY antibody (Dianova) and Alexa Fluor 568-conjugated anti-mouse-IgG antibody (Invitrogen, USA, CA), diluted 1∶40 and 1∶250, respectively. For negative controls, immunohistochemistry was performed using chicken preimmune serum and irrelevant mouse IgG1 antibody (Immunotools). Nuclear DNA of the cells was stained with 4′,6-diamidine-2-phenylindole-dihydrochloride (DAPI, Sigma-Aldrich, USA). Fluorochrome-labeled antibodies and DAPI-stained nuclei were evaluated under a fluorescence microscope (Axioskop, Zeiss, Jena, Germany), original magnification×40 (objective lense Plan-Neofluar, numerical aperture 0.75), following published protocols [43]. To confirm the hydrophobic nature of the target of the anti-Gb3Cer/CD77 antibodies, lipids were extracted with methanol and chloroform/methanol (1/1, v/v) from the mounted sections prior to staining.

### Infrared Matrix-assisted Laser Desorption/Ionization Orthogonal Time-of-Flight (IR-MALDI-o-TOF) Mass Spectrometry

The specifications of the IR-MALDI-o-TOF mass spectrometer (MDS Sciex, Concord, Ontario, Canada) and direct TLC-IR-MALDI-MS-analysis from immunopositive bands of the Stx1 TLC overlay assay have been detailed by Distler et al. [34]. GSLs were analyzed in the positive ion mode using glycerol as matrix.

### Statistical Analysis

Primary densitometric data of immunostained Gb3Cer/CD77 bands and secondary densitometric data, obtained from difference values of matched pairs calculated by subtracting normal from related tumor tissue values, were compiled with the software package SPSS, version 14.0.2 (SPSS Inc., Chicago, IL) for nonparametric statistical analysis. The sign test was used for the set of primary densitometric data to examine the significance of relationship between Gb3Cer/CD77 expression and neoplastic transformation. The median test was performed for primary and secondary densitometric data to test whether Gb3Cer/CD77-expression was significantly different among the tumors comprising various clinicopathologic categories. Correlation coefficients were calculated for ranked values of the primary densitometric data of normal and tumor tissues, respectively, and the ranked difference values. Kendall's τ was used to assess the degree of association between Gb3Cer/CD77-expression and histopathological grading. All tests were two tailed and αwas set at 0.05.

## Results

**Figure 1:**
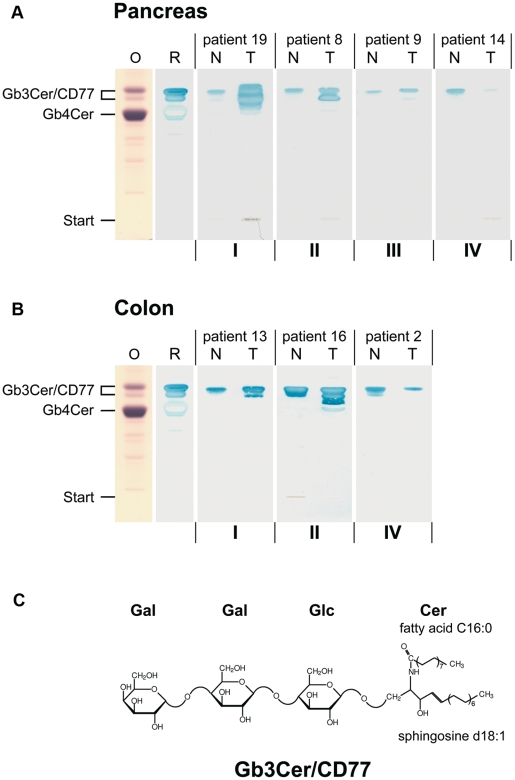
TLC overlay assay detection of tumor-associated Gb3Cer/CD77 in pancreas (A) and colon cancer (B). Aliquots from crude lipid extracts equivalent to 2 mg tissue wet weight of normal (N) and tumor tissue (T) were simultaneously separated by TLC. Neutral GSLs from human erythrocytes served as positive reference in the overlay assay (R, 10.8 µg) and the orcinol stain (O, 16.0 µg). The synopsis of Gb3Cer/CD77-expression in cancerous tissues of both tumor entities is provided in Table 1, and the histopathological data of pancreas and colon carcinomas are summarized in Supplementary Tables S1 and S2, respectively. Representative examples of pancreas (A) and colon cancers (B) of tumor categories I to IV are shown. None of the investigated colon carcinomas was found with equal expression of Gb3Cer/CD77 in adjacent normal tissue (B). Thus, category III remained vacant in the cohort of patients. C, structure of Stx1 receptor Gb3Cer (d18:1, C16:0). The ceramide (Cer) lipid anchor preferentially appears in healthy human tissues with C24 or C16 fatty acid but constant sphingosine (d18:1).

We probed tissue from 21 pancreatic and 16 colon adenocarcinoma patients for expression of Stx-receptor Gb3Cer/CD77 (for structure see Figure 1). For this purpose, crude lipid extracts and cryosections from postoperative matched pairs of malignant *versus* adjacent unaffected tissues from the same patients were analyzed by means of TLC overlay technique and immunofluorescence microscopy. To gain further structural details of tumor-associated modifications of Gb3Cer/CD77 species, a novel mass spectrometry approach was employed that combines TLC immunodetection with IR-MALDI-o-TOF-MS [8], [34]. The wet weights of the investigated cancerous and healthy tissues and the corresponding pathology data are listed in Supplementary Tables S1 and S2.

### TLC Immunodetection of Tumor-Associated Gb3Cer/CD77 in Pancreas and Colon Cancer

Identical aliquots of crude lipid extracts of matched pairs from neoplastic and corresponding unaffected tissues were subjected simultaneously to TLC, followed by TLC overlay detection of Gb3Cer/CD77 using Stx1 and the anti-Gb3Cer/CD77 antibody. Positive bands were ranked according to the different expression of Gb3Cer/CD77; rank 1 indicates the highest intensity (Table 1). Based on densitometry, tumors were grouped into four categories: ranging from high (I), and moderate (II) overexpression, equal (III), and lowered expression (IV).

**Table 1:** Expression of Gb3Cer/CD77-GSLs in pancreatic and colon carcinomas.

**Pancreas cancer**				
**Category** *	**%**	**Rank** †	**Grading** ‡	**Patient**
**I**	**28.6**	1	3	19
		2	2	10
		3	2–3	12
		4	3	4
		5	3	18
		6	2	3
**II**	**33.3**	7	2	13
		8	2	8
		9	3	7
		10	2	20
		11	2	17
		12	2	16
		13	2	5
**III**	**14.3**	14	2	21
		15	2	9
		16	3	1
**IV**	**23.8**	17	2	15
		18	1	2
		19	X	6
		20	1–2	11
		21	2	14
**Colon cancer**				
**Category** *	**%**	**Rank** †	**Grading** ‡	**Patient**
**I**	**18.8**	1	2	6
		2	2	8
		3	2	13
**II**	**62.5**	4	3	7
		5	3	11
		6	2	16
		7	2–3	15
		8	3	4
		9	2	12
		10	3	1
		11	2	10
		12	2–3	9
		13	2	5
**IV**	**18.8**	14	2	2
		15	2	14
		16	3	3

* Based on the secondary densitometric data, tumors were grouped into categories I to IV corresponding to high (I) and moderate (II) overexpression, equal (III) and lowered expression (IV).

† The 21 pancreatic tumors were ranked from 1 to 21 and the 16 colon tumors from 1 to 16, whereby rank 1 indicates the highest, rank 2 the second highest, etc., and the last rank the lowest expression of Gb3Cer/CD77.

‡ Histopathologic grading: G1, well differentiated; G2, moderately differentiated; G3, poorly differentiated; G4, undifferentiated; GX, histopathologic grading cannot be assessed.

#### Pancreatic Cancers

Figure 1A shows four single representative Stx1 TLC overlay stains of tumor and related healthy tissue extracts, each representing one of the four categories of pancreatic cancers. In total, 61.9% of the investigated pancreatic carcinomas showed moderate to high overexpression of Gb3Cer/CD77, whereas 14.3% exhibited equal and 23.8% diminished Gb3Cer/CD77 expression levels (see Table 1). Globotetraosylceramide (Gb4Cer), known as “low affinity” ligand of Stx1, was undetectable in pancreatic tumors and adjacent normal tissues using Stx1 as detection tool. TLC immunostains employing the polyclonal anti-Gb3Cer/CD77 antibody were similar in findings to those obtained with the Stx1 TLC overlay assays showing little higher sensitivity in tracing of Gb3Cer/CD77-species (see Supplementary Figure S1A).

#### Colon Cancers

Figure 1B shows three single representative Stx1 TLC overlay stains of colonic cancer matched pairs, each representing one of the three categories verified in colonic cancers. Gb3Cer/CD77 was moderately to highly overexpressed in 81.3% of malignant *versus* healthy colon tissues. None of the colon tumors exhibited equal expression resulting in vacant category III. Less Gb3Cer/CD77 was detected in 18.8% of the tumor tissue (see Table 1). As in pancreatic tissues, Gb4Cer was undetectable in colon tumors and adjacent normal tissues using Stx1 as detection tool. The parallel TLC immunostains employing the polyclonal anti-Gb3Cer/CD77 antibody are shown in Supplementary Figure S1B.

### Immunohistochemical Detection of Tumor-Associated Gb3Cer/CD77 in Pancreas and Colon Cancer

Binding of anti-Gb3Cer/CD77 antibody to frozen sections of pancreas and colon carcinoma tissues and to corresponding healthy tissues of the same patients was monitored histochemically. Sections were co-immunostained with an antibody against the endothelial cell marker CD31, and cell nuclei were visualized with DAPI fluorochrome.

**Figure 2:**
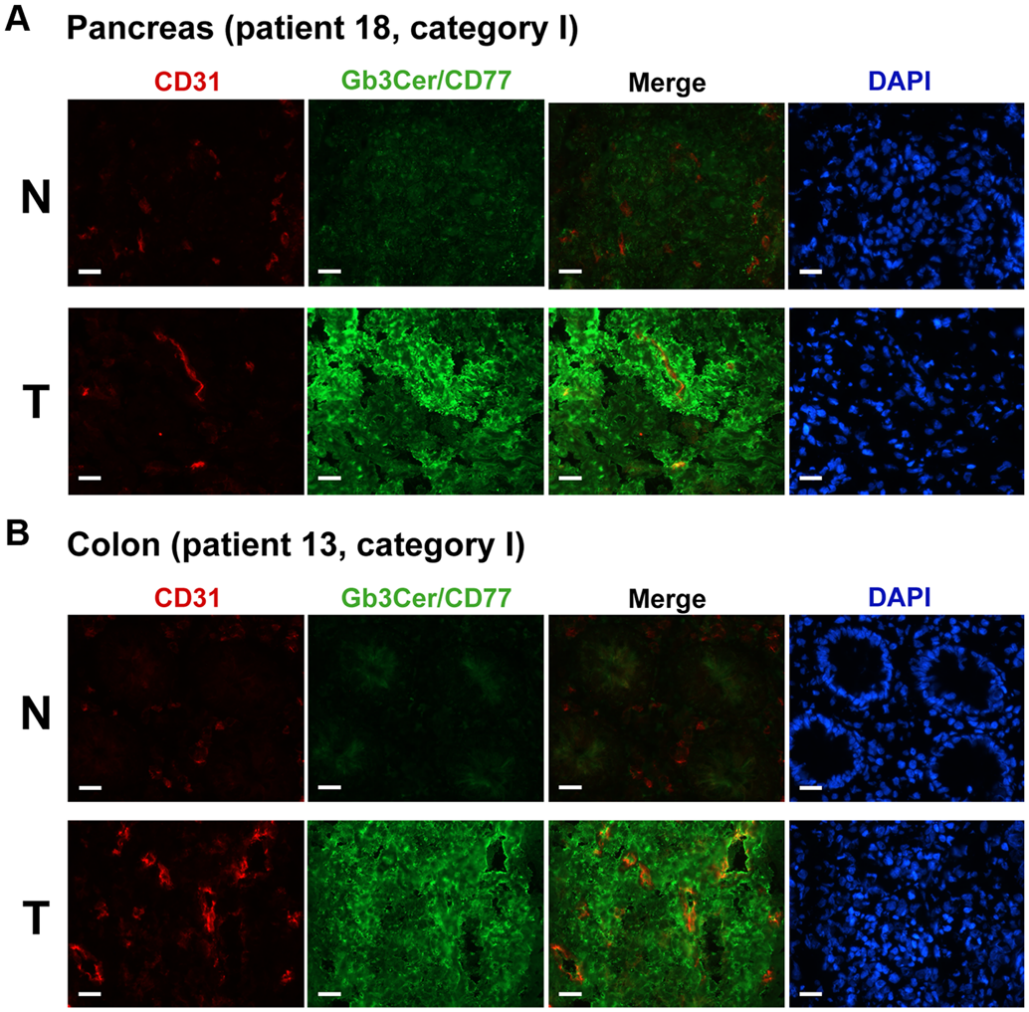
Immunohistological detection of Gb3Cer/CD77 in cryosections of pancreas (A) and colon cancer (B). Sections of normal (N) and tumor tissue (T) of pancreatic cancer patient 18 (A) and of colon cancer patient 13 (B) were immunostained with the anti-Gb3Cer/CD77 antibody and coimmunostained with an antibody against the endothelial cell marker CD31. Cell nuclei were detected with DAPI. Scale bars correspond to 20 µm. Representative examples of tumors of category I are shown (see Table 1). The histopathological data of pancreas and colon carcinoma are summarized in Supplementary Tables S1 and S2, respectively.

For example, extensive antibody binding to pancreatic tumor cells (T) was detected for patient 18 (tumor category I) as shown in Figure 2A. In contrast, little or no Gb3Cer/CD77 was found in normal pancreatic tissue (N). Occasional endothelial cells of the blood vessels, known to carry Gb3Cer/CD77 [21], [39], [43], showed also positive staining in pancreatic malignant tissue.

Immunohistochemical comparisons demonstrate high Gb3Cer/CD77 expression levels in colon tumors (T) as exemplified for patient 13, Figure 2B. In contrast, adjacent normal tissue (N) from the same patient lacked Gb3Cer/CD77. Angiogenesis seems to be advanced in some investigated colon cancers as evidenced by CD31 and Gb3Cer/CD77 positive endothelial cells within the tumor mass.

Control sections incubated with mouse IgG1 isotype controls as well as with chicken preimmune serum did not stain. After GSL extraction of the tissues, Gb3Cer/CD77 was no longer detectable with the antibody (data not shown).

### Structural Characterization of Tumor-Associated Gb3Cer/CD77 in Pancreas and Colon Cancer

The Stx1 positive bands of TLC-separated lipid extracts were directly analyzed on the TLC plates by TLC-IR-MALDI-MS as described [34]. Mass spectra were acquired in the positive ion mode and all Gb3Cer/CD77 species were detected as singly charged monosodiated molecular ions.

#### Pancreatic Cancers

**Figure 3:**
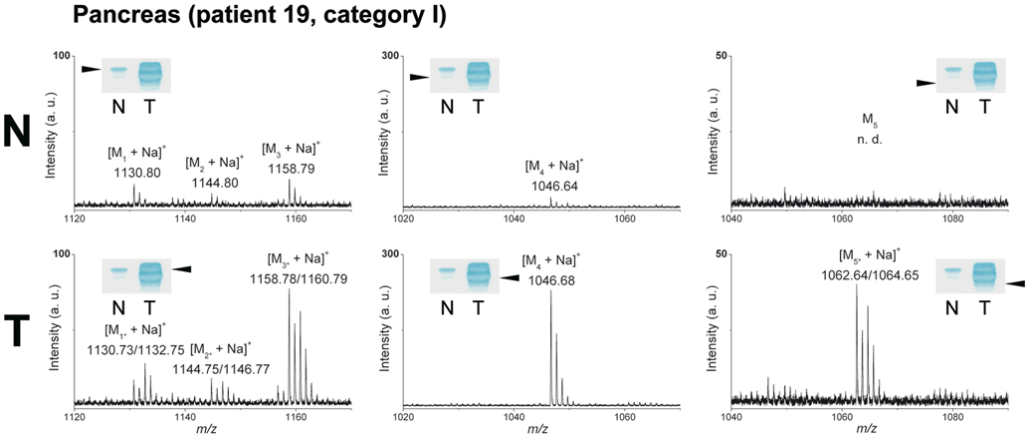
Tracing tumor-associated Gb3Cer/CD77 species in pancreas cancer. Lipid extracts equivalent to 2 mg wet weight of normal (N) and tumor tissue (T) of patient 19 (tumor category I, see Table 1 and Supplementary Table S1) were simultaneously separated by TLC followed by overlay analysis using Stx1. The Stx1 positive Gb3Cer/CD77 bands, which are marked with arrowheads in the insets, were analyzed by TLC-IR-MALDI-MS. The prevalent [M_3*_+Na]^+^ molecular ions of the upper band of the tumor tissue (T, left panel) correspond to Gb3Cer (d18:1/d18:0, C24:0) and the minor ions could be assigned to Gb3Cer (d18:1/d18:0, C23:0) and Gb3Cer (d18:1/d18:0, C22:0), respectively. In the upper band of normal tissue (N, left panel) much lower intensities of these molecular ions were observed. The high abundant [M_4_+Na]^+^ ions of the lower band in the tumor (T, middle panel) represent Gb3Cer (d18:1, C16:0), which is detectable only in trace quantities in normal tissue (N, middle panel). Analysis of the lowest band of the tumor (T, right panel) revealed [M_5*_+Na]^+^ ions, which are indicative for hydroxylated Gb3Cer (d18:1/d18:0, h16:0). This Gb3Cer/CD77 variant was undetectable in normal tissue (N, right panel). The *m/z* values of molecular ions and proposed structures of Gb3Cer/CD77-variants of malignant and adjacent normal tissues as well as references from human erythrocytes are listed in Supplementary Table S3.

Combined Stx1 detection and TLC-IR-MALDI-MS demonstrate individual compositional changes of overexpressed Gb3Cer/CD77 species in neoplastic compared to unaffected pancreatic tissue (e.g., patient 19 (tumor category I), Figure 3). Analyzing the upper of the Stx1 positive triple band of the tumor (T, left panel, inset), most abundant [M_3*_+Na]^+^ molecular ions point to the presence of Gb3Cer (d18:1/d18:0, C24:0) with saturated d18:0 sphinganine besides the more common monounsaturated d18:1 sphingosine (sphingenine). The same holds for the minor [M_2*_+Na]^+^ and [M_1*_+Na]^+^ ions, which refer to the structures of Gb3Cer (d18:1/d18:0, C23:0) and Gb3Cer (d18:1/d18:0, C22:0), respectively. In contrast, only low expression of Gb3Cer/CD77 could be detected in the upper Stx1 positive band of normal pancreatic tissue as shown in Figure 3 (N, left panel). The high abundance [M_4_+Na]^+^ ions of the lower Stx1 positive band of the tumor (T, middle panel) indicate strong overexpression of Gb3Cer (d18:1, C16:0) in tumor tissue compared to very low abundance ions in the corresponding healthy tissue (N, middle panel). However, the most prominent structural alteration was found in the lowest Stx1 positive band of the cancerous tissue by detecting [M_5*_+Na]^+^ ions (T, right panel), which were undetectable in normal tissue (N, right panel). When compared to Gb3Cer (d18:1, C16:0) (T, middle panel), the shift in 16 atomic mass units indicates hydroxylation of the ceramide moiety that leads to the proposed structure of Gb3Cer (d18:1, h16:0) carrying a hydroxylated C16:0 fatty acid. In addition, ions at *m/z* 1064.65 indicate the presence of Gb3Cer (d18:0, h16:0). This striking ceramide modification occurred in 85.7% of the pancreas tumors so far investigated. A synopsis of the proposed structures of Gb3Cer/CD77 species is provided in the Supplementary Table S3.

#### Colon Cancers

**Figure 4:**
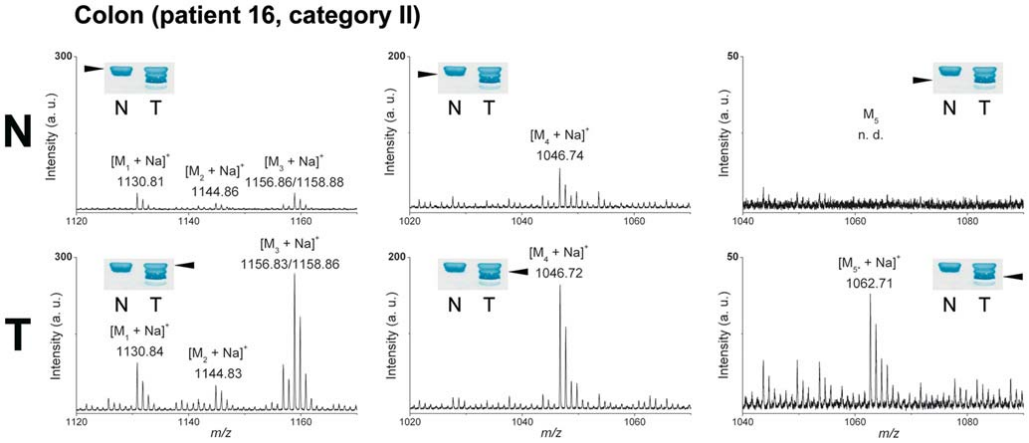
Tracing tumor-associated Gb3Cer/CD77 species in colon cancer. Lipid extracts equivalent to 2 mg wet weight of normal (N) and tumor tissue (T) of patient 16 (tumor category II, see Table 1 and Supplementary Table S2) were simultaneously separated by TLC followed by overlay analysis using Stx1. The Stx1 positive Gb3Cer bands, which are marked with arrowheads in the insets, were analyzed by TLC-IR-MALDI-MS. The prevalent [M_3_+Na]^+^ molecular ions of the upper band of the tumor tissue (T, left panel) correspond to Gb3Cer (d18:1, C24:1/C24:0) and the less abundant [M_2_+Na]^+^ and [M_1_+Na]^+^ ions to Gb3Cer (d18:1, C23:0) and Gb3Cer (d18:1, C22:0), respectively. In the upper band of normal tissue (N, left panel) much lower intensities of corresponding molecular ions were observed. The strong [M_4_+Na]^+^ ions of the lower band in the tumor (T, middle panel) represent Gb3Cer (d18:1, C16:0), which is detectable only in low quantities in normal tissue (N, middle panel). Analysis of the lowest band of the tumor (T, right panel) revealed [M_5*_+Na]^+^ ions, which are indicative for hydroxylated Gb3Cer (d18:1, h16:0). This Gb3Cer/CD77 variant was undetectable in normal tissue (N, right panel). The *m/z* values of molecular ions and proposed structures of Gb3Cer/CD77-variants of malignant and adjacent normal tissues as well as references from human erythrocytes are listed in Supplementary Table S3.

Stx1 detection combined with TLC-IR-MALDI-MS allowed us to profile the individual structures of overexpressed Gb3Cer/CD77 species as exemplarily shown for patient 16 in Figure 4 (T, left panel). High abundant [M_3_+Na]^+^ ions point to Gb3Cer (d18:1, C24:1/C24:0) as the most likely Gb3Cer/CD77 variants in the upper Stx1 positive band, accompanied by less pronounced [M_2_+Na]^+^ and [M_1_+Na]^+^ ions, which correspond to Gb3Cer (d18:1, C23:0) and Gb3Cer (d18:1, C22:0), respectively. The same ions but with much lower intensities were obtained from the upper Stx1 positive band of the normal tissue (N, left panel). In the lower band of the tumor (T, middle panel), prominent [M_4_+Na]^+^ ions could be assigned to the Gb3Cer (d18:1, C16:0) structure, which is also detectable in the normal tissue but with less ion intensity (N, middle panel). In the lowest band [M_5*_+Na]^+^ ions indicate the presence of the Gb3Cer (d18:1, h16:0) variant with hydroxylated C16:0 fatty acid, which could not be found in the normal tissue (N, right panel). This remarkable ceramide modification was found in approximately 19% of the colon tumor TLC immunostains so far investigated. A synopsis of the proposed structures of Gb3Cer/CD77 species is provided in the Supplementary Table S3.

### Statistical Analysis

**Figure 5:**
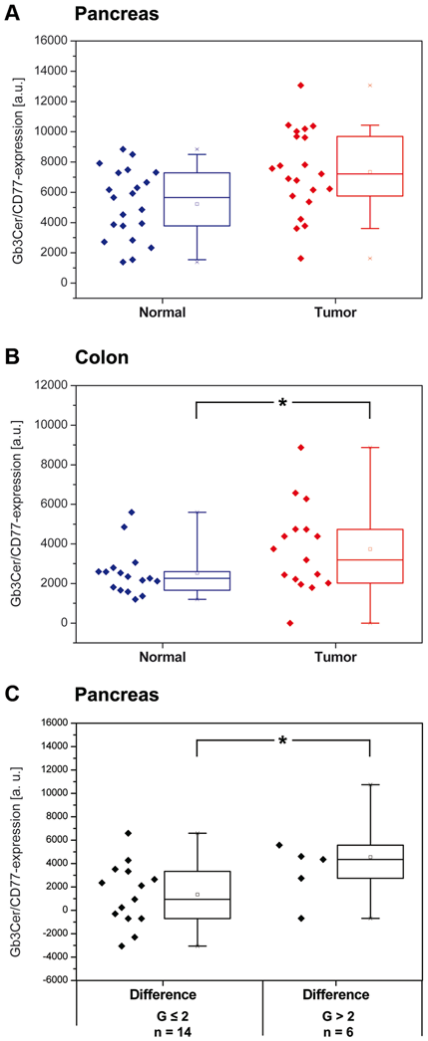
Box plots of Gb3Cer/CD77 expression in pancreas (A) and colon (B) normal and tumor tissues. The boxes are bounded above and below by the 25% and 75% percentiles. The lines in the boxes and the little squares indicate the median and mean values and the two×the minimum and maximum values, respectively. A, although not significant (P = 0.189, n = 21), an enhanced expression of Gb3Cer/CD77 could be observed in pancreatic tumors compared to normal tissues. B, the expression of Gb3Cer/CD77 is markedly increased in tumor colon tissues compared to that in the adjacent normal tissues indicating an association between Gb3Cer/CD77 expression and neoplastic transformation (*, P = 0.021, n = 16). C, correlation between Gb3Cer/CD77 expression and pancreatic tumor differentiation (n = 20 patients, because one tumor could not be assigned; see Table 1). Tumors were divided into two groups according to histopathologic grading: grade≤2 (n = 14 patients) and grade>2 (n = 6 patients). Rank correlation analysis of difference values points to a statistically significant higher Gb3Cer/CD77 expression in pancreas tumors graded as>2 (*, P = 0.039, τ = 0.396).

Box plots of the primary densitometric data of TLC overlay assay determined Gb3Cer/CD77 levels in the pancreatic (n = 21 patients) and colon (n = 16 patients) tissues are provided (Figure 5A and B, respectively). Overexpression of Gb3Cer/CD77 recorded in 81.3% of colon cancer compared to adjacent healthy tissues was significant (P = 0.021), whereas increased expression of Gb3Cer/CD77 in 61.9% of malignant pancreatic tissues was not significant (P = 0.189). In both pancreatic and colon cancer, the presence of Stx-detected Gb3Cer/CD77 bands did not correlate to any clinicopathological parameters (for pathological data see Supplementary Tables S1 and S2). However, for pancreatic tumors, the difference values of Gb3Cer/CD77 expression correlate with the grade of tumor differentiation (P = 0.039, τ = 0.396) indicating higher levels of Gb3Cer/CD77 in less differentiated malignancies. This association between poor differentiation of tumors in patients with Gb3Cer/CD77 overexpression in malignant pancreatic tissue is depicted in the box plots of Figure 5C. In conclusion, the expression of Gb3Cer/CD77 is greater in less differentiated malignant tissue suggesting the Stx receptor as a potential marker for poor differentiation of pancreatic tumors.

## Discussion

By clustering in lipid raft membrane microdomains [11], [44], the oligosaccharide chains of the GSLs exhibit strong binding activities towards their ligands [45]. Hence, tumor-associated GSLs, i.e., GSLs showing elevated expression in cancerous compared to normal tissues, are perfectly suited for tumor diagnosis and targeted anticancer therapies [31], [46], [47]. Consequently, antibodies, lectins or toxins with GSL binding specificities have engendered great interest in oncology. For example, two novel monoclonal antibodies, SC104 [6] and 14F7 [48], were recently raised against the oligosaccharide epitopes of tumor-associated sialylated GSLs ( = gangliosides) suggesting certain gangliosides as molecular targets for antibody-based cancer immunotherapy. In addition to monoclonal antibodies, plant and bacterial toxins that bind to GSLs are currently under investigation for clinical purposes. Examples are the ribosome-inactivating proteins (RIPs) viscumin from the plant *Viscum album* [49], which is specific for CD75s-gangliosides [5] and Stxs from Stx-producing *E. coli* (STEC) [9], [33]. Stxs, which are unique among bacterial toxins in sharing rRNA-*N*-glycosidase activity with plant RIPs [50], satisfy several principal challenges in oncology like molecular stability, and capabilities to cross physiologic barriers, and to withstand extracellular or intracellular degradation [32].

Stx escapes intracellular degradation after cell surface binding and retrograde transport, which depends largely on the molecular arrangement of GSLs in the plasma membrane. Only GSLs that associate strongly with lipid rafts carry AB_5_ toxins retrogradely and sort the toxins to the endoplasmic reticulum [51]. Although the terminal Galα1-4Gal-disaccharide is the primary recognition domain, the chain length of the fatty acid of the ceramide moiety might be important not only for the affinity of Stx1 towards Gb3Cer/CD77 [52] but also for the retrograde sorting of the toxin [53]. A more efficient intracellular routing of Stx to the endoplasmic reticulum occurs when the toxin is associated with Gb3Cer/CD77 variants carrying shorter chain fatty acids (primarily C16), which correlates with higher cellular sensitivity. Substitution of Gb3Cer/CD77-species with longer chain fatty acids (C22, C24) on the other hand results in retrograde transport to the Golgi apparatus only [53]. This lipoform-dependent traffic of Gb3Cer/CD77 from the cell surface to the endoplasmic reticulum has been hypothesized as a new signal transduction pathway. Importantly, most pancreatic and colon tumors showed up-regulation of Gb3Cer/CD77-species with C16:0 fatty acids, which supposedly correlate with augmented sensitivity to Stx1 induced cytotoxicity [53]. Interestingly, we observed additional hydroxylation in the ceramide moiety of more than 80% of pancreas and in ca. 20% of colon tumor derived Gb3Cer/CD77, most likely due to biosynthesis of h16:0 fatty acid, which has limited distribution in healthy tissues. In earlier studies hydroxylated fatty acids were found prominently in association with neoplastic transformation of human tissue in ceramides of fucosylated GSLs [54], [55] and the association of hydroxyl fatty acids with ceramides containing both C18 sphingosine and C18 phytosphingosine in a Lewis^x^-containing ganglioside has been described in great detail [56]. In order to elucidate the functional importance, Gb3Cer/CD77 fatty acid hydroxylation augments the binding affinity of Stx [57], rendering hydroxylated Gb3Cer/CD77 a highly efficient target for oncological purposes. An enhanced content of Gb3Cer/CD77 carrying hydroxylated fatty acids has been detected in ovarian carcinoma-derived cells, which survived selectively in the presence of taxol and cisplatin. This modification of the GSL structure occurred in association with anticancer-drug resistance [58]. Further on another study provided evidence for elevated levels of Gb3Cer/CD77 in primary ovarian cancers and hypersensitivity to Stx1 of multidrug resistant tumor-derived cell lines [26], [53]. All these data suggest an exceptionally attractive perspective for the direct targeting of Gb3Cer/CD77-expressing tumors employing Stx or Stx-derived constructs as a therapeutic for patients with unresectable tumors or advanced cancer refractory to chemotherapy.

The expression of Gb3Cer/CD77 in the neovasculature adjacent to and within the tumor has been previously observed for primary ovarian carcinomas and their metastases [26], astrocytomas and neuroblastomas [53]. As tumors depend on the generation of new vasculature to proliferate, targeting neovascular cells is another approach to inhibit tumor growth that might “starve” tumors as cancer cells are deprived of blood flow. Indeed, Lingwood et al. [53] showed the complete long-term elimination of human astrocytoma xenografts in nude mice after Stx1 administration. Massive apoptosis was observed in both tumor and vascular cells within the treated xenograft, suggesting tumor elimination not only by antineoplastic but also by antiangiogenic activity.

Consistent with recently published data [9], we observed increased expression of Gb3Cer/CD77 in malignant colon tissues. However, expression was not correlated to parameters such as tumor differentiation or stage grouping. Importantly, for pancreatic tumors, which exhibited markedly increased Gb3Cer/CD77 in most neoplasms (61.9%), we could determine correlation with the grade of tumor differentiation, demonstrating a trend towards augmented Gb3Cer/CD77 in less differentiated malignancies. Thus, overexpression in pancreatic tumors suggests Stx receptors as potential markers for poor differentiation in this type of cancer. Of note, Falguières et al. observed that nonmetastasized and metastasized colon tumors from Gb3Cer/CD77-positive primary colon tumors retained their Gb3Cer/CD77 profile and binding capacity towards the Stx B-subunit [9].

In conclusion, our studies suggest that Stx-targeting of Gb3Cer/CD77 expressing malignancies provide an opportunity to develop novel strategies to treat pancreatic and colon cancer. In light of enhanced Gb3Cer/CD77 expression on human cancer cells, it is tempting to propose the use of Stx and Stx-constructs for tumor cell delivery purposes to aid the recovery of unresectable or metastatic as well as chemoresistant tumors.

## Supporting Information

Table S1 Pathological data of pancreatic carcinoma and wet weights of normal and malignant tissues of the pancreas.
(0.07 MB DOC)

Table S2 Pathological data of colonic carcinomas and wet weights of normal and malignant tissues of the colon.
(0.06 MB DOC)

Table S3 Detected molecular ions and proposed structures of Gb3Cer/CD77 variants obtained by direct TLC-IR-MALDI-MS from healthy and malignant tissues from pancreas and colon together with references from human erythrocytes.
(0.06 MB DOC)

Figure S1 TLC overlay assay detection of tumor-associated Gb3Cer/CD77 in pancreas (A) and colon cancer (B). Aliquots from crude lipid extracts equivalent to 1 mg (pancreas) and 0.5 mg tissue wet weight (colon) of normal (N) and tumor tissue (T) were simultaneously separated by TLC and subjected to anti-Gb3Cer antibody TLC overlay assay. Neutral GSLs from human erythrocytes served as positive reference in the overlay assay (R, 10.8 µg) and the orcinol stain (O, 16.0 µg). Patients were assigned to 4 tumor categories according to the different expression of Gb3Cer/CD77 in malignant versus healthy tissues: I, high overexpression; II, moderate overexpression; III, equal expression, and IV, lowered expression. The synopsis of Gb3Cer/CD77-expression in cancerous tissues of both tumor entities is provided in Table 1, and the histopathological data of pancreas and colon carcinomas are summarized in Supplementary Tables S1 and S2, respectively. Representative examples of pancreas (A) and colon cancers (B) of tumor categories I to IV are shown. None of the investigated colon carcinomas was found with equal expression of Gb3Cer/CD77 in adjacent normal tissue (B). Thus, category III remained vacant in the cohort of patients.
(6.83 MB TIF)
