# Supplementary material for: Shiga Toxin Receptor Gb3Cer/CD77: Tumor-Association and Promising Therapeutic Target in Pancreas and Colon Cancer
Source: PLoS One. 2009 Aug 28;4(8):e6813. doi: 10.1371/journal.pone.0006813 (PMC2730034; doi:10.1371/journal.pone.0006813)
Supplement: Table S1 — Pathological data of pancreatic carcinoma and wet weights of normal and malignant tissues of the pancreas. (0.07 MB DOC) [file pone.0006813.s001.doc]

Table S1: Pathological data of pancreatic carcinoma and wet weights of normal and malignant tissues of the pancreas.

| **Patient** | **pT^*^** | **pN^†^** | **cM^‡^** | **UICC stage^§^** | **G^║^** | **Tissue wet weight [mg]** | |
| --- | --- | --- | --- | --- | --- | --- | --- |
|  |  |  |  |  |  | **Normal** | **Tumor** |
| 1 | 3 | 0 | 0 | IIA | 3 | 24.1 | 32.1 |
| 2 | 3 | 1 | 1 | IV | 1 | 23.2 | 27.1 |
| 3 | 3 | 1 | 1 | IV | 2 | 44.1 | 75.4 |
| 4 | 3 | 1 | 0 | IIB | 3 | 58.3 | 85.4 |
| 5 | 3 | 1 | 0 | IIB | 2 | 20.2 | 40.4 |
| 6 | 3 | 1 | 0 | IIB | X | 55.8 | 74.9 |
| 7 | 3 | 1(2/8) | 0 | IIB | 3 | 42.3 | 59.7 |
| 8 | 2 | 1 | 0 | IIB | 2 | 110.9 | 59.1 |
| 9 | 3 | 0 | 0 | IIA | 2 | 63.8 | 53.2 |
| 10 | 4 | 1(4/8) | 0 | III | 2 | 143.5 | 153.0 |
| 11 | 3 | 0 | 0 | IIA | 1-2 | 13.9 | 154.0 |
| 12 | 3 | 1 | 0 | IIB | 2-3 | 32.5 | 71.2 |
| 13 | 4 | 1(9/24) | 0 | III | 2 | 77.6 | 119.6 |
| 14 | 3 | 1 | 1 | IV | 2 | 110.1 | 153.4 |
| 15 | 3 | 1(2/20) | 0 | IIB | 2 | 27.7 | 88.3 |
| 16 | 3 | 1(2/19) | 0 | IIB | 2 | 18.0 | 27.6 |
| 17 | 3 | 1 | 0 | IIB | 2 | 60.4 | 54.7 |
| 18 | 3 | 1(8/27) | 0 | IIB | 3 | 28.4 | 45.1 |
| 19 | 3 | 1(2/10) | 0 | IIB | 3 | 92.6 | 40.6 |
| 20 | 3 | 0(0/17) | 0 | IIA | 2 | 12.8 | 77.9 |
| 21 | 3 | 0(0/11) | 0 | IIA | 2 | 197.5 | 75.8 |

**^*^**T = The extent of the primary tumor. T0: No evidence of primary tumor, Tis: Carcinoma in situ; T1: Tumor limited to the pancreas, Ø ≤ 2 cm, T2: Tumor limited to the pancreas, Ø > 2 cm, T3: Tumor extends directly into any of the following: duodenum, bile duct, peripancreatic tissues, T4: Tumor extends directly into any of the following: stomach, spleen, colon, adjacent large vessels

**^†^** N = The absence or presence and extent of regional lymph node metastasis. N0: No regional lymph node metastasis, N1: Regional lymph node metastasis

**^‡^** M = The absence or presence of distant metastasis. M0: No distant metastasis, M1: Distant metastasis

**^§^** Stage grouping. Stage 0: Tis N0 M0, Stage IA: T1 N0 M0, Stage IB: T2 N0 M0, Stage IIA: T3 N0 M0, Stage IIB: T4 N0 M0/ T1 N1 M0/ T2 N1 M0/ T3 N1 M0, Stage III: T4 N1 M0, Stage IV: Any T any N M1

**^║^** Histopathological grading. G1: well differentiated, G2: moderately differentiated, G3: poorly differentiated, G4: undifferentiated, GX: Histopathological grading cannot be assessed
