# Supplementary material for: Shiga Toxin Receptor Gb3Cer/CD77: Tumor-Association and Promising Therapeutic Target in Pancreas and Colon Cancer
Source: PLoS One. 2009 Aug 28;4(8):e6813. doi: 10.1371/journal.pone.0006813 (PMC2730034; doi:10.1371/journal.pone.0006813)
Supplement: Table S2 — Pathological data of colonic carcinomas and wet weights of normal and malignant tissues of the colon. (0.06 MB DOC) [file pone.0006813.s002.doc]

Table S2: Pathological data of colonic carcinomas and wet weights of normal and malignant tissues of the colon.

| **Patient** | **pT^*^** | **pN^†^** | **cM^‡^** | **UICC stage^§^** | **G^║^** | **Tissue wet weight [mg]** | |
| --- | --- | --- | --- | --- | --- | --- | --- |
|  |  |  |  |  |  | **Normal** | **Tumor** |
| 1 | 3/4 | 2 | 0 | IIIC | 3 | 19.6 | 12.6 |
| 2 | 3 | 2 | 1 | IV | 2 | 86.1 | 51.8 |
| 3 | 3 | 0 | 0 | IIA | 3 | 62.4 | 24.9 |
| 4 | 4 | 2 | 1 | IV | 3 | 56.5 | 47.4 |
| 5 | 2 | 0 | 0 | I | 2 | 99.5 | 22.6 |
| 6 | 4 | 0 | 0 | IIB | 2 | 59.0 | 13.8 |
| 7 | 3 | 1 | 0 | IIIB | 3 | 23.5 | 96.0 |
| 8 | 2 | 2 | 0 | IIIC | 2 | 112.10 | 11.2 |
| 9 | 3 | 1 | 0 | IIIB | 2-3 | 104.8 | 14.7 |
| 10 | 3 | 0 | 0 | IIA | 2 | 82.3 | 24.2 |
| 11 | 3 | 2 | 1 | IV | 3 | 73.2 | 31.8 |
| 12 | 4 | 1 | 1 | IV | 2 | 161.8 | 32.4 |
| 13 | 3 | 0 | 0 | IIA | 2 | 116.9 | 157.1 |
| 14 | 1 | 1 | 1 | IV | 2 | 78.7 | 71.8 |
| 15 | 4 | 1 | 0 | IIIB | 2-3 | 52.3 | 73.9 |
| 16 | 3 | 0 | 0 | IIA | 2 | 41.2 | 21.8 |

**^*^**T = The histopathological extent of the primary tumor. T0: No evidence of primary tumor, Tis: Carcinoma in situ (intraepithelial or invasion of the lamina propria), T1: Tumor invades submucosa, T2: Tumor invades muscularis propria, T3: Tumor invades through the muscularis propria into the subserosa or into nonperitonealized pericolic or perirectal tissues, T4: Tumor directly invades other organs or structures and/or perforates visceral peritoneum

**^†^** N = The histopathological absence or presence and extent of regional lymph node metastasis. N0: No regional lymph node metastasis, N1: Metastasis in one to three regional lymph nodes, N2: Metastasis in four or more regional lymph nodes

**^‡^** M = The clinical and radiological absence or presence of distant metastasis. M0: No distant metastasis, M1: Distant metastasis

**^§^** Stage grouping according to UICC. Stage 0: Tis N0 M0, Stage I: T1 N0 M0/T2 N0 M0, Stage IIA: T3 N0 M0, Stage IIB: T4 N0 M0, Stage IIIA: T1 N1 M0/T2 N1 M0, Stage IIIB: T3 N1 M0/T4 N1 M0, Stage IIIC: Any T N2 M0, Stage IV: Any T any N M1

**^║^** Histopathological grading. G1: well differentiated, G2: moderately differentiated, G3: poorly differentiated, G4: undifferentiated
