# Supplementary material for: Shiga Toxin Receptor Gb3Cer/CD77: Tumor-Association and Promising Therapeutic Target in Pancreas and Colon Cancer
Source: PLoS One. 2009 Aug 28;4(8):e6813. doi: 10.1371/journal.pone.0006813 (PMC2730034; doi:10.1371/journal.pone.0006813)
Supplement: Table S3 — Detected molecular ions and proposed structures of Gb3Cer/CD77 variants obtained by direct TLC-IR-MALDI-MS from healthy and malignant tissues from pancreas and colon together with references from human erythrocytes. (0.06 MB DOC) [file pone.0006813.s003.doc]

**Table S3: Detected molecular ions and proposed structures of Gb3Cer/CD77 variants obtained by direct TLC-IR-MALDI-MS from healthy and malignant tissues from pancreas and colon together with references from human erythrocytes**.

| **Proposed structure** |  | ***m/z* (monoisotopic)**  **Detected^†^**  **Pancreas, Colon** | ***m/z* (monoisotopic)**  **Calculated** |
| --- | --- | --- | --- |
|  |  |  |  |
| **Pancreas (P) and Colon (C)^*^** |  |  |  |
|  |  |  |  |
| Normal tissue |  |  |  |
|  |  |  |  |
| Gb3Cer/CD77 (d18:1, C24:0) | P, C | 1158.79, 1158.88 | 1158.79 |
| Gb3Cer/CD77 (d18:1, C24:1) | C | 1156.86 | 1156.77 |
| Gb3Cer/CD77 (d18:1, C23:0) | P, C | 1144.80, 1144.86 | 1144.77 |
| Gb3Cer/CD77 (d18:1, C22:0) | P, C | 1130.80, 1130.81 | 1130.75 |
| Gb3Cer/CD77 (d18:1, C16:0) | P, C | 1046.64, 1046.74 | 1046.66 |
|  |  |  |  |
| Malignant tissue |  |  |  |
|  |  |  |  |
| Gb3Cer/CD77 (d18:0, C24:0) | P | 1160.79 | 1160.80 |
| Gb3Cer/CD77 (d18:1, C24:0) | P, C | 1158.78, 1158.86 | 1158.79 |
| Gb3Cer/CD77 (d18:1, C24:1) | C | 1156.83 | 1156.77 |
| Gb3Cer/CD77 (d18:0, C23:0) | P | 1146.77 | 1146.79 |
| Gb3Cer/CD77 (d18:1, C23:0) | P, C | 1144.75, 1144.83 | 1144.77 |
| Gb3Cer/CD77 (d18:0, C22:0) | P | 1132.75 | 1132.77 |
| Gb3Cer/CD77 (d18:1, C22:0) | P, C | 1130.73, 1130.84 | 1130.75 |
| Gb3Cer/CD77 (d18:1, C16:0) | P, C | 1046.68, 1046.72 | 1046.66 |
| Gb3Cer/CD77 (d18:0, h16:0) | P | 1064.65 | 1064.67 |
| Gb3Cer/CD77 (d18:1, h16:0) | P, C | 1062.64, 1062.71 | 1062.66 |
|  |  |  |  |
| **Human erythrocytes^‡^** |  |  |  |
|  |  |  |  |
| Gb3Cer/CD77 (d18:1, C24:0) |  | 1158.81 | 1158.79 |
| Gb3Cer/CD77 (d18:1, C24:1) |  | 1156.79 | 1156.77 |
| Gb3Cer/CD77 (d18:1, C23:0) |  | 1144.78 | 1144.77 |
| Gb3Cer/CD77 (d18:1, C22:0) |  | 1130.77 | 1130.75 |
| Gb3Cer/CD77 (d18:1, C16:0) |  | 1046.68 | 1046.66 |

**^*^**Structures of Gb3Cer/CD77 species corresponding to Figure 3 (pancreas) and Figure 4 (colon). Detected Structures are listed according to their chromatographic properties.

**^†^** Mass spectra were acquired in the positive ion mode; all Gb3Cer/CD77 species were detected as singly charged [M + Na]^+^ molecular ions.

**^‡^** Reference Gb3Cer/CD77 from human erythrocytes [34, 40].
