# Supplementary figures and images for: Shiga Toxin Receptor Gb3Cer/CD77: Tumor-Association and Promising Therapeutic Target in Pancreas and Colon Cancer
Source: PLoS One. 2009 Aug 28;4(8):e6813. doi: 10.1371/journal.pone.0006813 (PMC2730034; doi:10.1371/journal.pone.0006813)

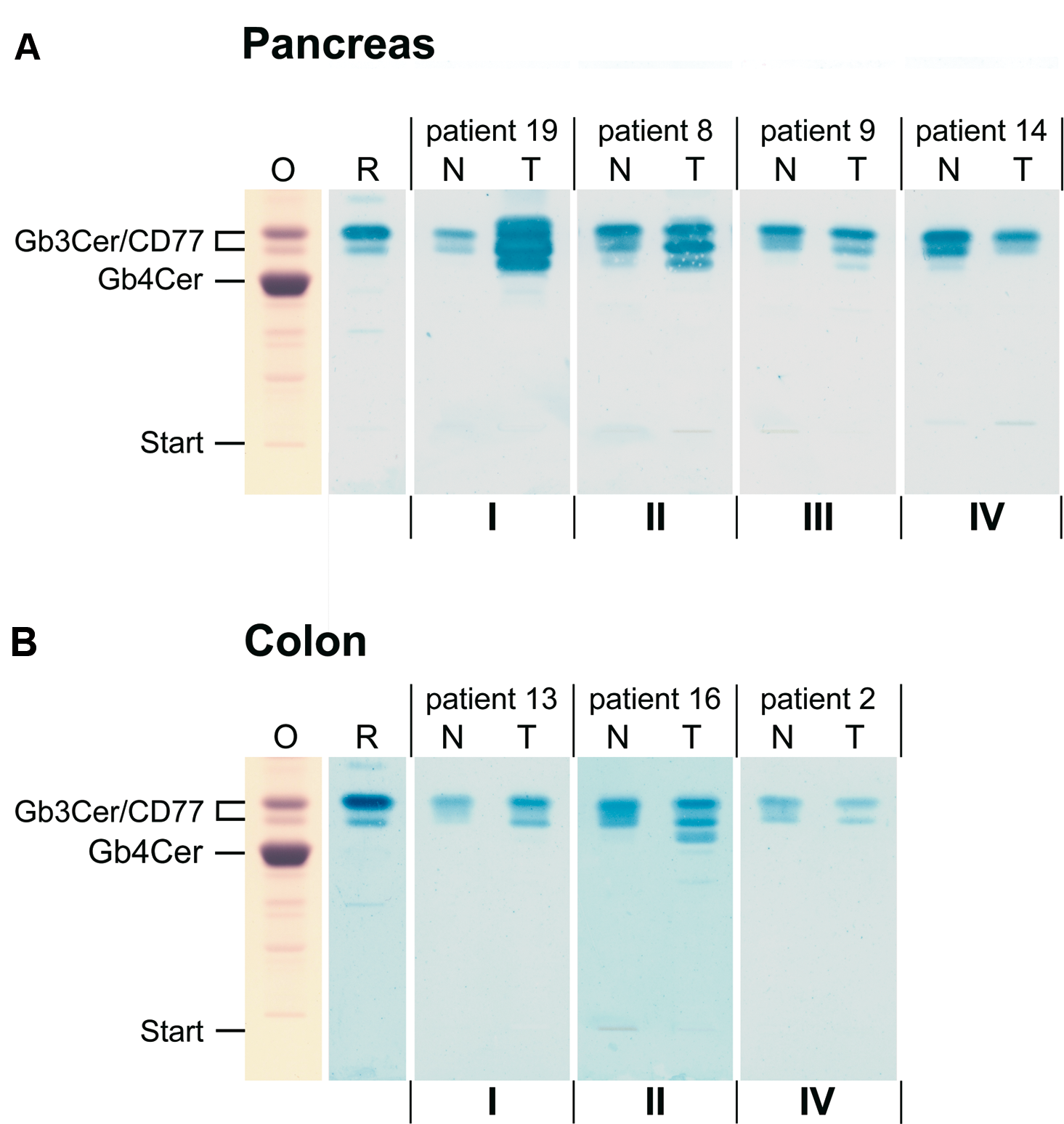

Supplement: Figure S1 — TLC overlay assay detection of tumor-associated Gb3Cer/CD77 in pancreas (A) and colon cancer (B). Aliquots from crude lipid extracts equivalent to 1 mg (pancreas) and 0.5 mg tissue wet weight (colon) of normal (N) and tumor tissue (T) were simultaneously separated by TLC and subjected to anti-Gb3Cer antibody TLC overlay assay. Neutral GSLs from human erythrocytes served as positive reference in the overlay assay (R, 10.8 µg) and the orcinol stain (O, 16.0 µg). Patients were assigned to 4 tumor categories according to the different expression of Gb3Cer/CD77 in malignant versus healthy tissues: I, high overexpression; II, moderate overexpression; III, equal expression, and IV, lowered expression. The synopsis of Gb3Cer/CD77-expression in cancerous tissues of both tumor entities is provided in Table 1, and the histopathological data of pancreas and colon carcinomas are summarized in Supplementary Tables S1 and S2, respectively. Representative examples of pancreas (A) and colon cancers (B) of tumor categories I to IV are shown. None of the investigated colon carcinomas was found with equal expression of Gb3Cer/CD77 in adjacent normal tissue (B). Thus, category III remained vacant in the cohort of patients. (6.83 MB TIF) [file pone.0006813.s004.tif]
